# Tools for studying growth patterns and chemical dynamics of aggregated *Pseudomonas aeruginosa* exposed to different electron acceptors in an alginate bead model

**DOI:** 10.1038/s41522-018-0047-4

**Published:** 2018-02-19

**Authors:** Majken Sønderholm, Klaus Koren, Daniel Wangpraseurt, Peter Østrup Jensen, Mette Kolpen, Kasper Nørskov Kragh, Thomas Bjarnsholt, Michael Kühl

**Affiliations:** 1 Department of Immunology and Microbiology, Faculty of Health and Medical Sciences, University of Copenhagen, Blegdamsvej 3B, DK-2200 Copenhagen N, Denmark; 2 Marine Biology Section, Department of Biology, University of Copenhagen, Strandpromenaden 5, DK-3000 Helsingør, Denmark; 3 Department of Chemistry, University of Cambridge, Lensfield Road, Cambridge, CB2 1EW UK; 4 Department of Clinical Microbiology 9301, Copenhagen University Hospital, Rigshospitalet, Juliane Maries Vej 22, Copenhagen, Denmark; 5 Climate Change Cluster, University of Technology Sydney, Broadway, NSW 2007 Australia

## Abstract

In chronic infections, bacterial pathogens typically grow as small dense cell aggregates embedded in a matrix consisting of, e.g., wound bed sludge or lung mucus. Such biofilm growth mode exhibits extreme tolerance towards antibiotics and the immune defence system. The bacterial aggregates are exposed to physiological heterogeneity and O_2_ limitation due to steep chemical gradients through the matrix, which is are hypothesised to contribute to antibiotic tolerance. Using a novel combination of microsensor and bioimaging analysis, we investigated growth patterns and chemical dynamics of the pathogen *Pseudomonas aeruginosa* in an alginate bead model, which mimics growth in chronic infections better than traditional biofilm experiments in flow chambers. Growth patterns were strongly affected by electron acceptor availability and the presence of chemical gradients, where the combined presence of O_2_ and nitrate yielded highest bacterial growth by combined aerobic respiration and denitrification.

## Chronic infection: effects of electron acceptors

Bacterial growth is strongly affected by levels and gradients of electron accepting chemicals such as nitrate ions and oxygen molecules. Thomas Bjarnsholt, Michael Kühl, and colleagues at the University of Copenhagen grew *Pseudomonas aeruginosa* bacteria in beads composed of a natural carbohydrate called alginate. This method models the growth patterns in chronic infections more effectively than conventional procedures. The model reflects the fact that chronic infections typically involve dense aggregates of cells embedded in materials such as wound debris or mucus. This renders the bacteria resistant to antibiotics and immune defences. The researchers investigated the effect of varying concentration gradients and flow patterns of several electron acceptors. Bacterial growth was strongly influenced by the distribution of the electron acceptors. Adding electron acceptors to treatment regimes may enhance the activity of antibiotics against chronic infections.

## Introduction

Bacterial biofilms are ubiquitous in most natural habitats, where they play an integral role in the cycling of elements. However, biofilms are also associated with a wide range of harmful effects ranging from biofouling of ship hulls and drainpipes to the formation of biofilms on medical implants and indwelling devices.^1^ In chronic infections such as diabetic and venous leg ulcers or in the lungs of patients suffering from the genetic disorder cystic fibrosis (CF),^2,3^ biofilms grow as small dense cell aggregates devoid of surface association,^4,5^ in a matrix of exopolymeric substance consisting of polysaccharides, proteins, and eDNA.^6–9^ Such bacterial aggregates are embedded in a secondary matrix composed of, e.g., wound bed sludge or CF lung mucus. Bacterial aggregates exhibit physiological heterogeneity due to steep chemical gradients forming through the secondary matrix and into the biofilm.^10,11^ In particular, molecular oxygen (O_2_) has been shown to reach hypoxic and anoxic levels within the outer 50–100 µm of biofilms and chronic wounds.^12–14^ Steep O_2_ gradients are also a recognised feature of chronic infections,^13,15–17^ where activated polymorphonuclear leucocytes persistently accumulate around the bacterial aggregates^4,18^ leading to strong depletion of O_2_ due to formation of reactive oxygen species.^19,20^ Such complexity of the chemical landscape is believed to result in heterogeneous growth patterns, and the establishment of bacterial subpopulations exhibiting particular metabolic activities.^21^ This in turn can have an impact on the efficacy of antibiotic treatment as several studies have shown that O_2_ limitation is correlated to increased antibiotic tolerance of biofilms.^10–12,22^

The opportunistic pathogenic bacterium *Pseudomonas aeruginosa* is a key model organism for the study of biofilm infections, and it has been isolated from both chronic wounds and chronically infected lungs of CF patients. In order to gain further insight to the biofilm mode of growth of *P. aeruginosa*, several optical methods can be applied for visualising growth patterns and biofilm structure. The standard method of confocal laser scanning microscopy (CLSM) of stained or fluorescently tagged *P. aeruginosa* provides very localised information on biomass distribution (at µm scale). In combination with quantitative peptide nucleic acid fluorescence in situ hybridisation (PNA-FISH)^21,23^ CLSM can also be applied to quantify the growth potential^21^ by treating bacterial cells with PNA-FISH probes specific for *P. aeruginosa* 16S rRNA.^24^ While providing high-resolution data on bacterial growth, this method is based on fixed samples and is therefore an invasive technique. When aiming to unravel structural biofilm properties at mesoscopic to macroscopic levels (10 µm—mm length scale), optical coherence tomography (OCT) is a suitable alternative imaging technique.^25^ OCT employs near-infrared radiation (NIR) and provides a non-invasive alternative to light microscopy, enabling high-resolution 3D scanning of larger (mm^3^ to cm^3^) biofilm volumes in near-real time.^26–28^ As previously mentioned, bacterial aggregates are exposed to chemical and physiological heterogeneity due to steep O_2_ gradients. To further elucidate this aspect, microsensors can be used to investigate the chemical environment in a minimal invasive fashion.^29^ Microsensors are available for several analytes including O_2_ and nitrous oxide (N_2_O), a key intermediate product of denitrification.^30^ Another approach is to use chemical imaging with optical sensors (either immobilised in sensor films or particles) to visualise the chemical microenvironments in biofilms.^31–34^

Biofilms are often studied in vitro using continuous flow cell systems, wherein biofilms are grown attached to a surface and can exhibit a variety of structural morphologies including mushroom-shaped structures.^35^ However, in CF lungs and chronic wounds *P. aeruginosa* grows in dense suspended aggregates separated by a secondary matrix and with no attachment to a solid substrate or surface.^4^ The typical growth mode and biofilm shapes observed in flow chambers are thus not representative of the observed in vivo growth patterns of biofilms associated with chronic infections. To better mimic the in vivo conditions of *P. aeruginosa* in chronic infections, we recently employed an alginate bead model,^14^ wherein the bacteria form dense, spatially segregated micro colonies similar in size and structure to *P. aeruginosa* aggregates observed ex vivo in lungs from CF patients^21^ and chronic wounds.^4^

*P. aeruginosa* can grow anaerobically by performing arginine fermentation or using alternative electron acceptors^36–38^ and there is increasing evidence that *P. aeruginosa* can utilise the high physiological NO_3_^−^ and NO_2_^−^ levels in the CF lungs to grow under O_2_ limitation by performing denitrification.^39,40^ In this study, we investigated *P. aeruginosa* aggregate growth in alginate beads with different O_2_ and NO_3_^−^ availability mimicking physiological conditions encountered in the chronic infections.^41,42^ The bacterial biomass, growth rate, and chemical microenvironment in the beads was characterised using a novel combination of OCT, CLSM, PNA-FISH, viable cell counts, O_2_ and N_2_O microsensor analysis, as well as first attempts of chemical O_2_ imaging. We found significant effects of electron acceptor availability on the growth pattern and metabolic activity of *P. aeruginosa* forming in vivo-like microcolonies in the alginate bead model, and discuss the implications of our results for better treatment of chronic infections.

## Results

### Optical coherence tomography

**Fig. 1:**
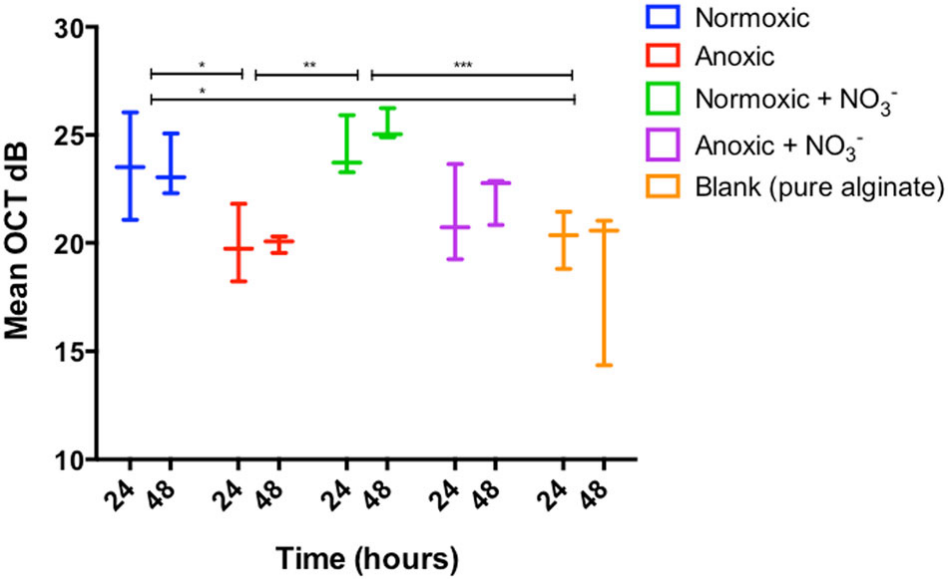
OCT signal intensity (dB) of alginate beads over 48 h of incubation. The OCT signal was averaged for an area covering a vertical depth of 150 μm (from the bead surface into the interior) and a lateral width of 100 μm and was used as a proxy for bacterial growth (see “Methods”). Bars represent average ± standard errors of the means from three replicates. Significant differences as indicated. *p > 0.05; **p ≤ 0.01; ***p ≤ 0.001

OCT facilitated non-invasive macroscopic imaging of the alginate beads (Supplementary Fig. S1A, B), wherein light scattering bacterial aggregates were identified by their high OCT signal. Due to dense growth of bacterial aggregates in the bead periphery, the vertical OCT signal attenuation was rapid and at about 200 µm below the bead surface the OCT signal approached that of the OCT signal of pure alginate (Supplementary Fig. S2). The OCT dB signal from the outermost peripheral 150 µm of the beads could thus be used as a proxy for bacterial biomass distribution. The OCT dB signal of anoxic beads (without NO_3_^−^) was similar to the blank control (without bacteria), thus suggesting no or very minor growth (Fig. 1). In contrast, the OCT signal from anoxic beads with NO_3_^−^ was significantly higher relative to the control after 48 h (*p* = 0.035). Normoxic beads and normoxic beads supplemented with NO_3_^−^ gave rise to the strongest OCT signal, indicative of intense growth and biomass accumulation in the bead periphery (Fig. 1, S1).

### Microscopy

**Fig. 2:**
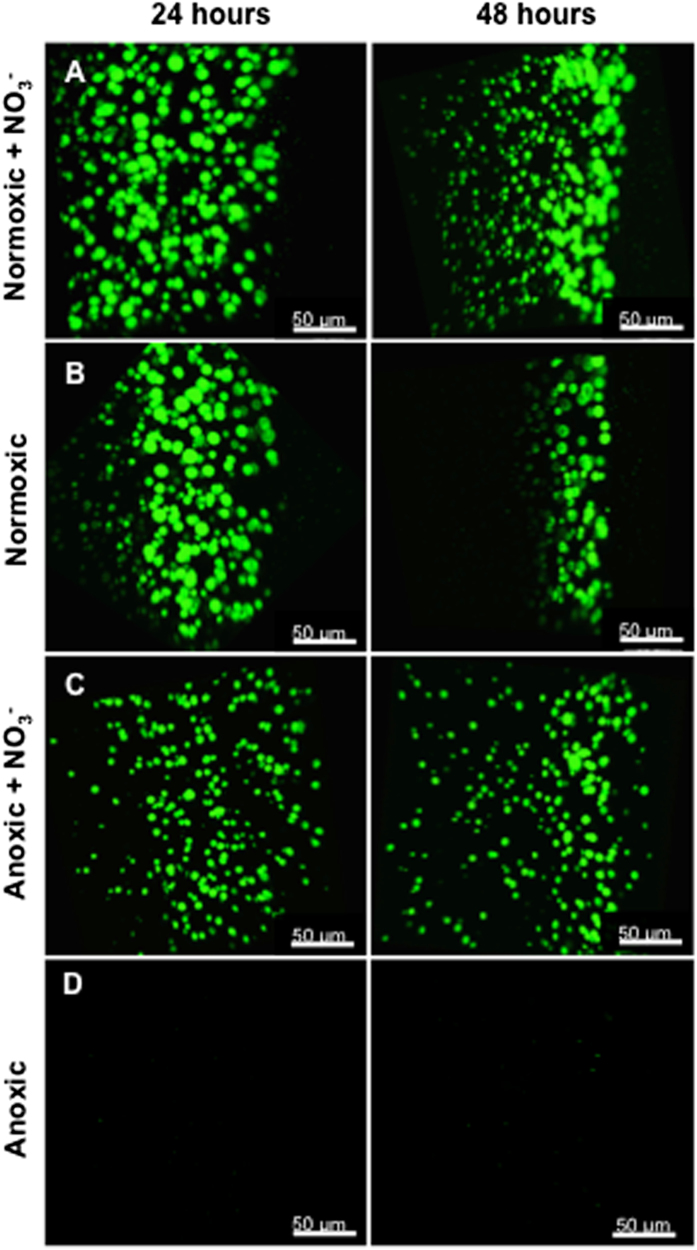
400× CLSM images of the growth dynamics of GFP-tagged *P. aeruginosa* grown under different conditions after 24 and 48 h. **a** Normoxic growth in the presence of NO_3_^−^ resulted in intense and deep growth with a tendency to form smaller aggregates in the deeper parts over time. **b** Normoxic growth resulted in peripheral growth over time. **c** Anoxic growth with NO_3_^−^ also supported growth, but with apparently smaller aggregates. **d** Anoxic growth without NO_3_^−^ did not support growth. The edge of the alginate bead is oriented to the right in the images. Size of scale bars: 50 μm

Bacterial growth and organisation in the alginate beads was visualised microscopically by CLSM using green fluorescent protein (GFP)-tagged *P. aeruginosa* (Fig. 2). In normoxic beads, *P. aeruginosa* showed peripheral growth (growth in the outermost ~100 µm of the bead) of bacterial microcolonies after 24 and 48 h (Fig. 2b). Normoxic growth in the presence of NO_3_^−^ resulted in a very intense growth, with larger aggregates in the periphery than observed under anoxic growth of *P. aeruginosa* in the presence of NO_3_^−^. Moreover, 48 h normoxic growth of *P. aeruginosa* with NO_3_^−^ resulted in a heterogeneous growth pattern, with bacterial microcolonies decreasing in size with distance from the surface of the alginate bead (Fig. 2a). Anoxic beads with NO_3_^−^ showed a homogeneous distribution of aggregate size in the bead after 24 h, but also exhibited signs of a more heterogeneous aggregate size distribution after 48 h growth with largest aggregates closer to the bead surface (Fig. 2c). No growth was observed in anoxic beads without alternative electron acceptors at 48 h (Fig. 2d), which was supported by the OCT imaging results.

### Microscopy combined with quantitative PNA-FISH

**Fig. 3:**
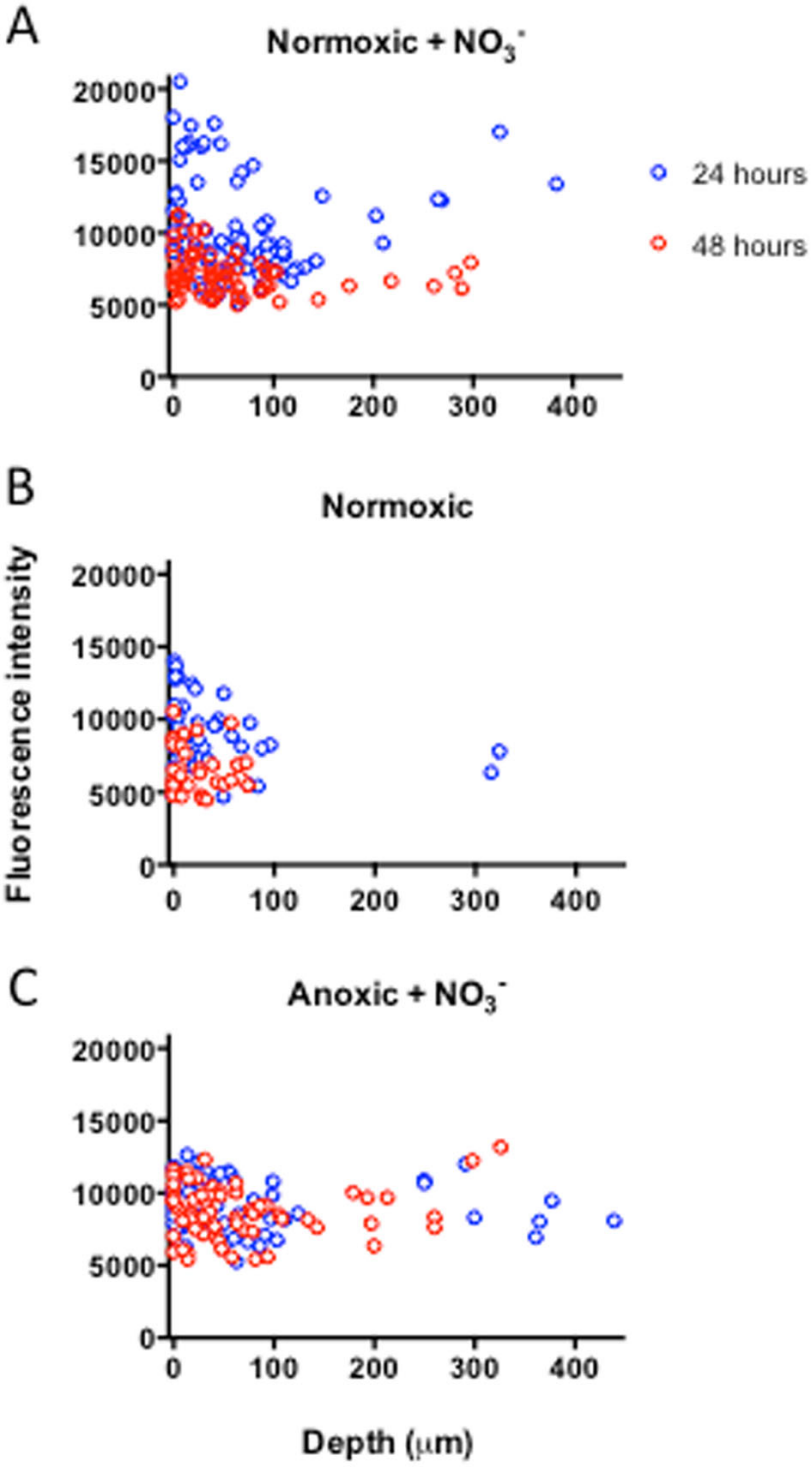
Growth potential of *P. aeruginosa* grown in alginate beads expressed as mean fluorescence intensity. **a** Normoxic growth conditions supplemented with NO_3_^−^, **b** normoxic growth conditions without NO_3_^−^, and **c** anoxic growth conditions in the presence of NO_3_^−^. *P. aeruginosa* was fluorescently labelled with Texas-Red-conjugated PNA-FISH probe tagging ribosomes, imaged by CLSM and quantification of intensity was performed by Imaris

The mean fluorescence intensity used here as a proxy for the growth potential (the maximum possible growth rate) of *P. aeruginosa*^21^ was quantified and plotted for each of the growth conditions. In normoxic beads, we found that NO_3_^−^ supplementation induced significantly deeper growth of *P. aeruginosa* aggregates (Fig. 3a). The median growth depth (interquartile range, IQR) was 51.6 (17.4–92.0) µm vs. 20.0 (2.0–45.0) µm (*p* < 0.0001) below the alginate bead surface. When NO_3_^−^ was present, the median growth depth (IQR) was comparable under normoxic vs. anoxic conditions, i.e., 51.6 (17.4–92.0) µm vs. 41.6 (14.2–88.6) µm (*p* = 0.481).

In normoxic beads with NO_3_^−^, the growth potential of *P. aeruginosa* was significantly higher after 24 h than after 48 h (10212 FU ± 3298 FU vs. 7141 FU ± 1470 FU; *p* < 0.001). Furthermore, we found a significantly higher growth depth at 24 h vs. 48 h, with a median growth depth (IQR) of 62.9 (25.9–98.0) µm vs. 38.8 (11.3–70.3) µm (*p* = 0.023) below the alginate bead surface (Fig. 3a). Growth depth in normoxic beads without NO_3_^−^ did not differ between the two time points (*p* = 0.139), but the growth potential, as estimated by mean fluorescence, was significantly higher at 24 h as compared to 48 h (9243 ± 2433 vs. 6586 ± 1614; *p* < 0.001) (Fig. 3b). In anoxic beads with NO_3_^−^, there was no significant difference in neither growth depth (*p* = 0.761) or in growth potential (*p* = 0.124) at 24 h vs. 48 h, and the growth potential remained high (9258 FU ± 1865 FU vs. 8750 FU ± 1780 FU) both after 24 and 48 h (Fig. 3c).

**Fig. 4:**
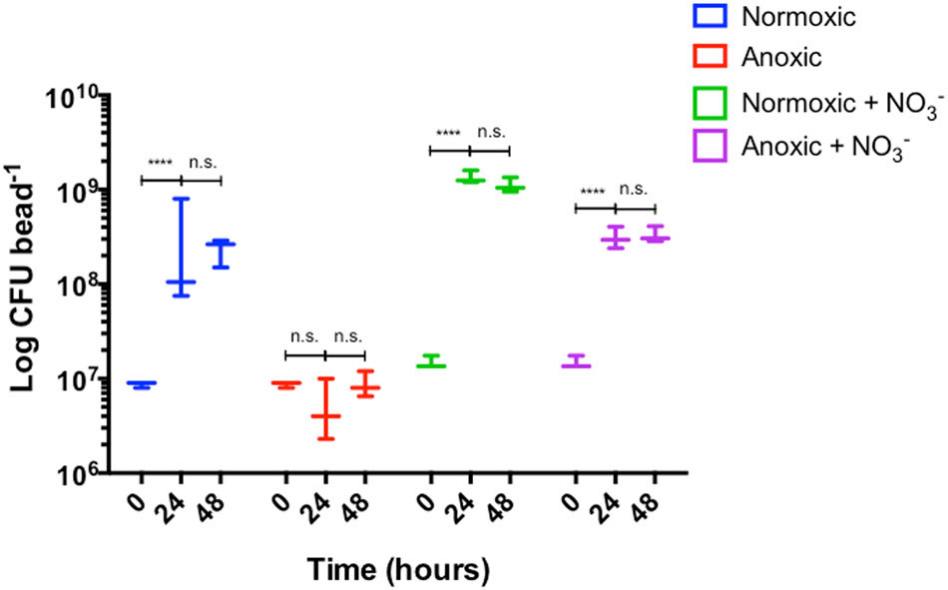
Viable cell counts as colony-forming units (CFU) from dissolved alginate beads after 0, 24, and 48 h. Highest CFU was observed for normoxic beads supplemented with NO_3_^−^. No change in CFU over time for the anoxic group was observed. Bars represent average ± standard errors of the means from three replicates. Significant differences as indicated. n.s. not significant; ****p ≤ 0.0001

When correlating growth potential to growth depth, we found no statistically significant correlation under any conditions. It was not possible to prepare paraffin slices for anoxic beads without NO_3_^−^, but as previously shown *P. aeruginosa* failed to grow under these conditions (Figs. 2, 4).

### Colony-forming unit (CFU)

All bead cultures were initiated with a CFU-defined cell density of ~10^7^ cells per bead (mean 1.175 × 10^7^; range 8.67 × 10^6^–1.483 × 10^7^) (Fig. 4). While CFU values remained stable in the anoxic culture without NO_3_^−^, the other growth conditions facilitated a significant increase in CFU of 1–2 log units from 0 to 24 h, which then remained constant between 24 and 48 h. The highest CFU was observed for the growth condition with both O_2_ and NO_3_^−^ available in the alginate beads (*p* < 0.0001).

### Microsensor measurements

**Fig. 5:**
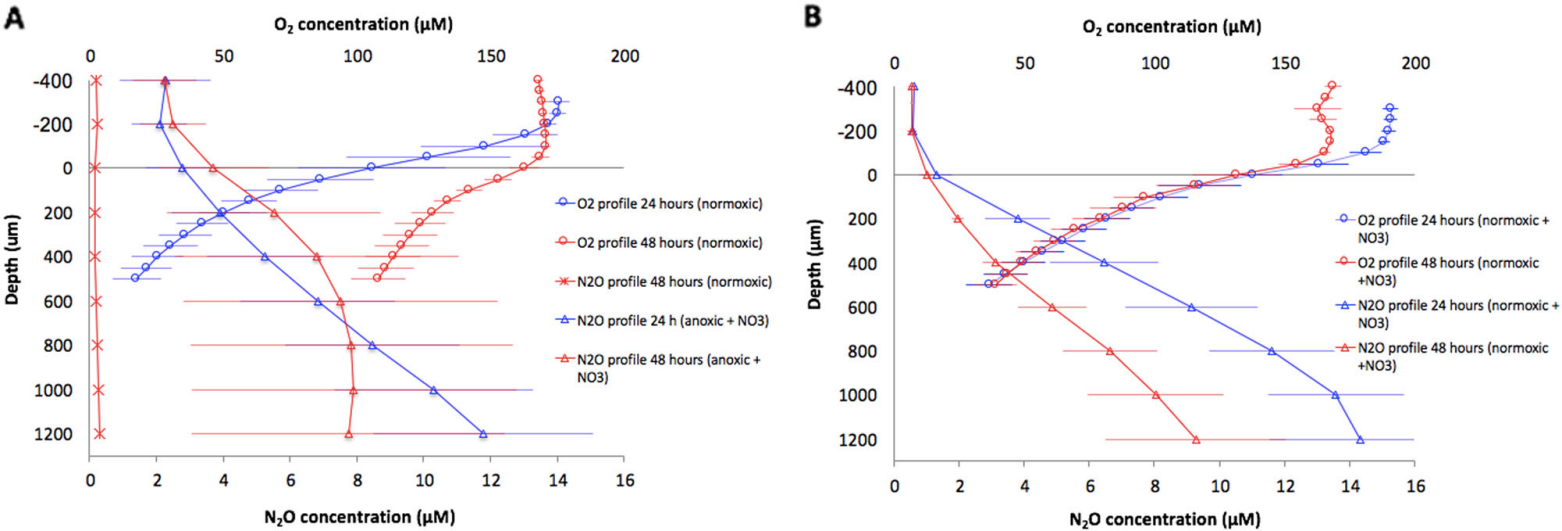
O_2_ and N_2_O profiles from alginate-encapsulated *P. aeruginosa*. **a** *P. aeruginosa* cultured under normoxic conditions (circles) and anoxic conditions with 10 mM NO_3_^−^ (triangles). Furthermore, a control measurement of N_2_O on alginate-encapsulated *P. aeruginosa* grown without NO_3_^−^ (asterisk). **b** *P. aeruginosa* cultured under normoxic conditions supplemented with 10 mM NO_3_^−^. O_2_ profiles (circles) and N_2_O profiles (triangles). Bars represent average ± standard deviation from 3 to 4 replicates. All measurements were performed at 37 °C in growth medium

**Fig. 6:**
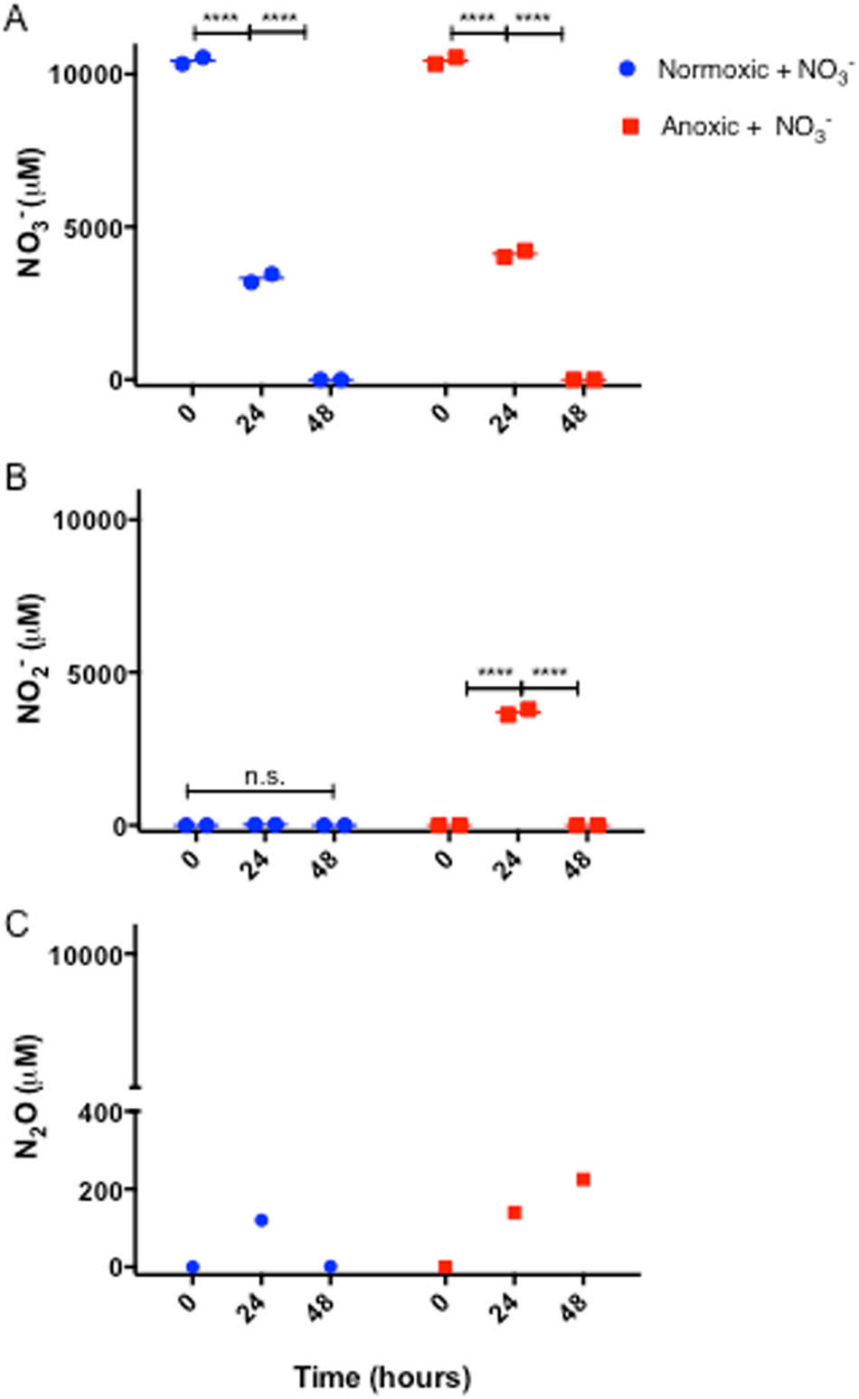
Dynamics of **a** NO_3_^−^, **b** NO_2_^−^, and **c** N_2_O (μM) in growth medium quantified by Griess colometric reaction and microsensor measurements directly in growth medium. NO_3_^−^ and NO_2_^−^ measurements are based on duplicate sampling from medium (error bars too small to be visualised), and N_2_O concentration represents on a single measurement directly in medium. Significant differences as indicated. n.s. not significant; ****p ≤ 0.0001

The O_2_ concentration profiles measured in normoxic alginate beads (without NO_3_^−^) after 24 h incubation showed the presence of an oxygenated zone in the peripheral 200 µm with a steep decline in the O_2_ concentration and an inner hypoxic zone (Fig. 5a). After 48 h, the slope of the profile decreased, indicating an overall decrease in O_2_ respiration rate from 24 to 48 h (Fig. 5a), leading to more O_2_ accumulation in the beads over time. No N_2_O production was detected in normoxic grown alginate beads without NO_3_^−^ (Fig. 5a). In normoxic alginate beads supplemented with NO_3_^−^, the maximal N_2_O concentration was detected in the lower, hypoxic zone (Fig. 6b). Contrary to normoxic beads (without NO_3_^−^), O_2_ profiles in NO_3_^−^ supplemented beads were stable between 24 and 48 h, whereas N_2_O production decreased (Fig. 6b). N_2_O profiles in anoxic beads with NO_3_^−^ revealed an increasing N_2_O concentration throughout the bead, with a slight decrease in production after 48 h (Fig. 6a).

### Fluxes of O_2_ and N_2_O

We found a stable O_2_ flux into the normoxic beads supplemented with NO_3_ (3.18 ± 0.66–3.19 ± 0.84 nmol O_2_ cm^−2^ min^−1^), and a tendency towards a decreasing O_2_ flux into normoxic beads (without NO_3_^−^) between 24 and 48 h (3.07 ± 1.33–1.84 ± 0.32 nmol O_2_ cm^−2^ min^−1^). Calculations of the efflux of N_2_O from the alginate beads revealed a stable N_2_O efflux in anoxic beads with NO_3_^−^ between 24 and 48 h (0.10 ± 0.03–0.13 ± 0.1 nmol N_2_O cm^−2^ min^−1^), whereas the normoxic beads with NO_3_^−^ showed a tendency towards a decrease in N_2_O efflux between 24 and 48 h (0.21 ± 0.06–0.09 ± 0.03 nmol N_2_O cm^−2^ min^−1^). None of the changes in flux over time were statistically significant.

### NO_3_^−^, NO_2_^−^, and N_2_O dynamics in growth medium

Measurements of NO_3_^−^ and NO_2_^−^ concentrations in the R2A growth medium supplemented with NO_3_^−^ showed that the initial concentration (*t* = 0) of NO_3_^−^ in the medium was 10.450 µM ± 148 µM (Fig. 6a), while no NO_2_^−^ could be detected (Fig. 6b). After 24 h, the NO_3_^−^ concentration had declined significantly to 4.119 ± 0.149 and 3.319 ± 0.191 µM (*p* < 0.0001) in the anoxic and normoxic cultures, respectively, and was completely depleted within 48 h (Fig. 6a). No NO_2_^−^ could be detected in the normoxic cultures, but in the anoxic cultures there was a significant accumulation of NO_2_^−^ after 24 h (*p* < 0.0001), which was depleted again after 48 h (Fig. 6b). When measuring the N_2_O concentrations directly in the growth medium with electrochemical sensors, we found no N_2_O in the growth medium without added NO_3_^−^ (data not shown). In the anoxic culture flasks with NO_3_^−^ addition (sealed airtight), denitrification led to an accumulation of N_2_O over time (Fig. 6c). After 24 h of incubation, a N_2_O concentration of 140 µM was measured in the medium, increasing to 225 µM after 48 h. In the normoxic culture with NO_3_^−^, N_2_O was measured at a concentration of 120 µM after 24 h, but only 1.5 µM after 48 h.

### O_2_ imaging

**Fig. 7:**
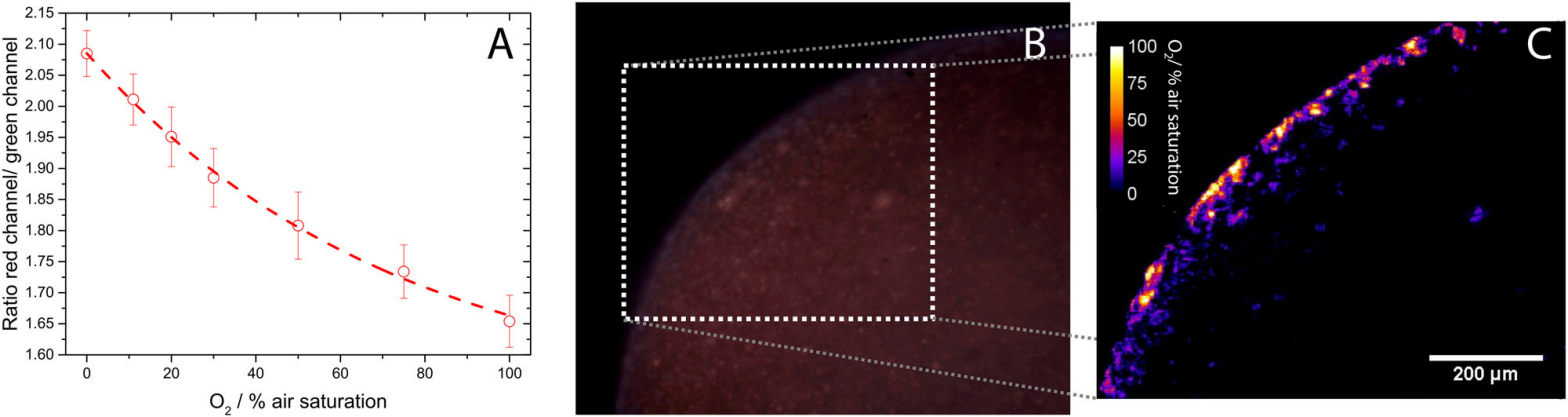
Ratiometric imaging of O_2_ with oxygen-sensitive nanoparticles incorporated in alginate beads with *P. aeruginosa*. **a** Calibration of nanoparticles in alginate beads submerged in water of increasing O_2_ concentrations. The O_2_ was quantified as the ratio of the Red and Green channels extracted from RGB colour images obtained with a simple USB microscope. **b** Alginate bead with sensor nanoparticles, **c** zoom-in and visualisation of O_2_ distribution in alginate bead after 24 h of growth with *P. aeruginosa*. Size of bar: 200 µm

The incorporation of O_2_-sensitive nanoparticles in the alginate beads enabled visualisation of the O_2_ distribution relative to the bacterial aggregates. The O_2_ images revealed steep O_2_ gradients in the periphery of the beads (Fig. 7c) forming a heterogeneous landscape of O_2_ concentration within the O_2_ provisioned part of the beads that roughly followed the aggregate distribution, where aggregates exhibited complete O_2_ depletion and hypoxia in the surrounding alginate matrix. Atmospheric O_2_ saturation was only observed at the immediate bead surface.

## Discussion

Cultures of *P. aeruginosa* in alginate beads exhibit several characteristics of aggregated bacteria found in the lungs of CF patients and in chronic wounds, in regard to aggregate size, growth, O_2_ limitation, physiological heterogeneity, and antibiotic tolerance.^14^ This enabled our study of in vivo-like growth patterns and the chemical microenvironment of *P. aeruginosa* with and without electron acceptors via a variety of invasive and non-invasive methods. The most intense growth of *P. aeruginosa* was observed in the presence of the two electron acceptors O_2_ and NO_3_^−^, enabling both aerobic respiration and denitrification in the alginate beads, while complete absence of electron acceptors resulted in an arrested growth state.

We studied the growth patterns of *P. aeruginosa* in the presence and absence of O_2_ and NO_3_^−^ at different spatial scales using OCT for mesoscopic and macroscopic scale, CLSM to unravel structures at a microscopic scale, and quantitative PNA-FISH for information on the growth potential. OCT has previously been used to study the formation and growth dynamics of biofilms in flow chambers and various carrier materials^43,44^ and of clinical biofilms in nasal polyps,^28^ but to our knowledge OCT has not previously been used to investigate bacteria growing in alginate beads. The presence of bacteria increased local backscatter in the alginate matrix resulting in a stronger OCT signal.^44^ However, the alginate matrix also scattered light, and a quantification of bacterial growth thus relies on a clear separation of the OCT signals originating from the bacteria and the alginate. We found that bacterial growth led to an enhancement of the OCT signal relative to the alginate bead, but only within the first 200 µm from the bead surface (see Supplementary Fig. S2). While OCT allows for a potential operational depth of view of several mm’s,^45^ the high density of bacteria in the outermost bead layers leads to intense multiple scattering of the NIR probing light causing a rapid vertical attenuation and thus loss of image contrast of the collected OCT signal^46^ resulting in an operational depth of view that was comparable to CLSM.^47^ Evidently, such application of OCT can be further optimised, e.g., by decreasing scatter in the alginate matrix using washed less optically opaque alginate, but such technical optimisation was beyond the scope of this study. Still, the OCT data in our study complemented higher-resolution microscopy techniques and provided a non-invasive macroscopic overview of the growth dynamics and growth zones in intact alginate beads. With OCT, it was thus possible to monitor the overall growth zone of *P. aeruginosa* in alginate beads (Fig. S1), while high-resolution CLSM enabled (i) visualisation and quantification of microcolony size and distribution in the alginate beads (Fig. 2) and (ii) local growth potential measurements when used in combination with a quantitative PNA-FISH protocol (Fig. 3).

Supplementing *P. aeruginosa* with two electron acceptors (O_2_ and NO_3_^−^) resulted in higher growth potentials (Figs. 2, 3) at 24 h as compared to 48 h incubation. Moreover, a heterogeneous growth pattern of *P. aeruginosa* was observed in the alginate beads after 48 h incubation with larger aggregates situated near the oxygenated bead surface. Physiological heterogeneity within biofilms is largely driven by the activity and biomass distribution of the bacteria in combination with mass transfer limitation by diffusion of electron acceptors, substrate and products of bacterial metabolism in the biofilm matrix and in the diffusive boundary layer between the mixed water phase and the biofilm.^11^ Typically, bacterial depletion of O_2_ in the biofilm periphery limits diffusion to deeper parts,^48^ and we found that such development of O_2_ concentration gradients in the alginate beads lead to a heterogeneous growth pattern over time, where only the bacteria positioned near the bead surface had sufficient access to the preferred electron acceptor O_2_. While anoxic grown *P. aeruginosa* with access to NO_3_^−^ displayed a very homogeneous growth pattern after 24 h, these beads also showed signs of a heterogeneous growth pattern after 48 h (Fig. 2). This shift was probably due to depletion of bead-incorporated NO_3_^−^, where after NO_3_^−^ was only available by diffusion from the growth medium. These results are in accordance with previous studies showing a heterogeneous distribution of bacterial aggregate sizes, with significantly larger aggregates in the periphery as compared to deeper in the beads after 48 h in normoxic grown alginate-encapsulated *P. aeruginosa* with and without 100 mM NO_3_^−^ supplement.^14^

The results from OCT and CLSM corresponded well with the quantitative PNA-FISH results in terms of showing increased growth depth in the presence of NO_3_^−^, while the availability of O_2_ did apparently not affect growth depth into the alginate beads. The latter finding confirms that O_2_ is strongly affected by diffusion limitation.^49^ In addition, quantitative PNA-FISH provided an estimate on the growth potential, expressed as fluorescence intensity, which has been shown to correlate with growth rate in *P. aeruginosa*.^21^ Contrary to growth depth, the growth potential was affected by the presence of O_2_, and we found a significantly higher growth potential with O_2_ at 24 h as compared to 48 h. This suggests that the contribution of O_2_ to the overall growth may be limited after 48 h, and that the preferred electron acceptor O_2_ initially facilitates a more intense growth burst than with NO_3_^−^, which correlates well with the low growth rate of *P. aeruginosa* observed in vivo in the mucus of chronically infected CF lungs.^21^ Furthermore, this is in accordance with a higher energy yield by O_2_ respiration as compared to denitrification.^50,51^ Cell counts further confirmed a more intense growth in the presence of both O_2_ and NO_3_^−^ (Fig. 4).

Interestingly, bacterial growth in beads with no electron acceptors did not result in a decrease in CFU but rather lead to a steady state in cell counts throughout the study, which is in support of *P. aeruginosa’s* remarkable ability for long-time survival.^52^ Previous studies have thus demonstrated that *P. aeruginosa* is capable of long-term anaerobic survival via fermentation of amino acids,^52–54^ which were present in the growth medium.

To gain further insight to factors governing the growth dynamics of *P. aeruginosa* in the beads as observed with OCT, CLSM, and quantitative PNA-FISH, we used microsensors to quantify chemical gradients in the alginate beads. Based on such measurements, we estimated oxic respiration from the net consumption of O_2_ and denitrification from the net production of N_2_O in the beads, respectively. The observed decline in O_2_ in the water just above the alginate bead surface showed the presence of a diffusive boundary layer around the bead,^55^ and microsensor measurements demonstrated the presence of steep O_2_ concentration gradients and thus high O_2_ consumption in beads incubated under normoxic conditions. However, the O_2_ uptake decreased with time (Fig. 5a) in line with our observation of a decreasing growth rate over time as determined by quantitative PNA-FISH (Fig. 3). This may indicate onset of substrate limitation for bacterial growth in the alginate beads.

Beads incubated in normoxic medium with NO_3_^−^ showed steep O_2_ concentration gradients, but contrary to beads grown without NO_3_^−^, the O_2_ uptake did not decrease with time (Fig. 5b). However, such sustained high O_2_ consumption was not reflected in higher growth potentials, which showed a decrease between 24 and 48 h of incubation (Fig. 3a). Some bacteria can perform so-called aerobic denitrification during hypoxic conditions in the presence of NO_3_^−^. Initially described by Robertson and Kuenen,^56^ aerobic denitrification can explain how NO_3_^−^ respiration may proceed in the presence of O_2_.^56^ In *P. aeruginosa* this ability is evidenced by the expression of overlapping gene sets depending on O_2_ concentration, where hypoxia triggers denitrification genes.^57^ Moreover, the ability to perform intra-aerobic respiration has been proposed, where NO_3_^−^ is reduced to NO, which is dismutated into N (nitrogen) and O_2_.^58^ Such formed O_2_ would in theory proceed in the aerobic respiration chain, which would then support high O_2_ consumption rates. However, we note that the presence of aerobic denitrification or intra-aerobic respiration remains to be demonstrated in *P. aeruginosa*. Maximal N_2_O concentration was detected in the hypoxic zone of the alginate beads, indicating that denitrification mainly occurred here (Fig. 5). Production of N_2_O decreased with time (Fig. 5b), which may reflect NO_3_^−^ depletion in the beads and the surrounding medium. This was supported by the complete exhaustion of NO_3_^−^ after 48 h (Fig. 6a).

In beads incubated in anoxic medium with NO_3_^−^, *P. aeruginosa* solely relied on denitrification for anaerobic respiration as indicated by the observed N_2_O production, which decreased over time (Fig. 5b), leading to the appearance of a more heterogeneous size distribution of bacterial aggregates in the beads (Fig. 2) resembling the growth pattern observed in normoxic beads. As N_2_O is a key intermediate in the denitrification metabolic pathway,^36^ a decreasing N_2_O flux could indicate the onset of NO_3_^−^ limitation within the beads after 48 h of growth, which was supported by our finding of complete consumption of NO_3_^−^ after 48 h (Fig. 6a). We also followed the fate of NO_2_^−^ in the growth medium (Fig. 6b), which showed a peak in NO_2_^−^ for anoxic grown beads after 24 h, and complete depletion after 48 h. In normoxic incubations (with NO_3_^−^), no increase in NO_2_^−^ was observed, but we speculate that such a peak may have occurred before the 24 h measurement, as NO_3_^−^ in the presence of O_2_ was shown to facilitate a more intense growth initially. The N_2_O measurements in the growth medium showed that N_2_O accumulated to very high levels in the sealed system after 48 h (Fig. 6c) further supporting a strong NO_3_^−^ consumption during the incubations.

While our microsensor measurements revealed insight to the overall dynamics of O_2_ and N_2_O gradients in the alginate beads when incubated under different electron acceptor availability, we note that such measurements cannot provide detailed insight to the heterogeneous chemical landscape of individual microcolonies in the alginate beads. In a first attempt to link microcolony heterogeneity to the distribution in O_2_, we used ratiometric O_2_ imaging in a 24 h old alginate bead supplemented with O_2_-sensitive nanoparticles and grown under normoxic conditions (Fig. 7c). Ratiometric O_2_ imaging showed pronounced local variation in the outermost 100 µm of the alginate bead. However, the used camera system for O_2_ imaging did not have enough spatial resolution to investigate co-localisation and enable correlations between microcolony size and O_2_ distribution. However, such combined biomass and O_2_ imaging^34^ in the alginate beads has a strong potential to further resolve links between the chemical microenvironment and growth of *P. aeruginosa* in the alginate bead. Such approach would require more measurements of O_2_ distribution with nanoparticles in the beads using higher-resolution microscopic imaging.^33,59^

**Fig. 8:**
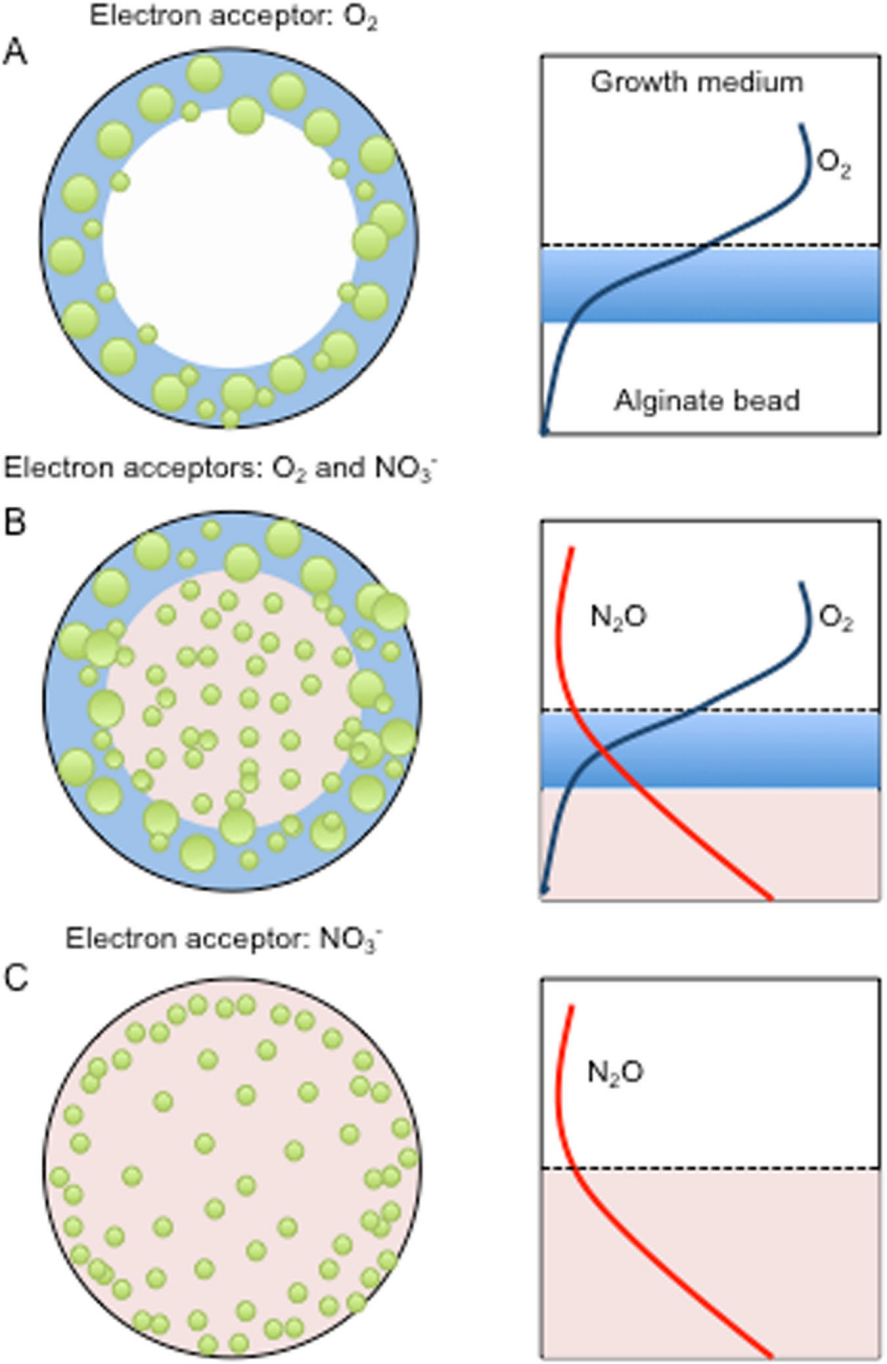
Schematic presentation of growth dynamics and chemical dynamics in alginate beads under different growth conditions. **a** Normoxic growth, **b** normoxic growth with NO_3_^−^, and **c** anoxic growth with NO_3_^−^. Green circles represent aggregates of *P. aeruginosa* within the alginate bead. O_2_: blue/light blue. NO_3_^−^: red/light red. Sizes not to scale

In conclusion, growth in alginate beads represents a useful in vitro model for the in vivo growth of *P. aeruginosa* in chronic infections. This model system is suitable for testing responses in bacterial metabolism and growth patterns to the availability of different electron acceptors and donors during chronic infections, and the effect of the different treatments are summarised in Fig. 8. The alginate bead model may also prove very suitable for testing antimicrobial susceptibility and tolerance of *P. aeruginosa* and other pathogens involved in mono-species and multi-species infections. It is well known that O_2_ limitation contributes to antibiotic tolerance of bacteria in biofilms,^22^ but addition of electron acceptors such as NO_3_^−^ and O_2_ may antagonise this effect and enhance the susceptibility towards antibiotics.^60,61^

## Methods

### Bacterial strains

A wild-type *P. aeruginosa* strain PAO1 was obtained from the *Pseudomonas* Genetic Stock Center (http://www.pseudomonas.med.ecu.edu). To enable visualisation of PAO1 cells, GFP constitutively expressed on plasmid pMRP9^62^ was used.

### Growth conditions

Cultures were propagated from −80 °C freeze culture stocks and grown overnight (ON) in lysogeny broth (LB) for approximately 18 h at 37 °C under continuous shaking at 180 RPM. Subsequently, the LB ON culture was used for inoculation in low nutritional R2A broth (Lab M Limited, UK) supplemented with 0.05 M Tris-HCl buffer (pH 7.6) and 0.5% glucose (abbreviated R2A), and left to acclimatise ON until further use. The medium to volume ratio was 1:2.5.

### Bead preparation

Alginate beads of 2.4 mm ± 0.1 mm in diameter (mean ± standard deviation) were prepared by a previously described method,^14^ with the exception of adding 10 mM potassium nitrate (KNO_3_^−^) (P8394, Sigma-Aldrich, USA) to some of the beads. In all experiments, beads were incubated in R2A medium (with or without 10 mM KNO_3_^−^) at 100 RPM at 37 °C for 24 or 48 h, respectively. For normoxic conditions, culture flasks were sealed with cotton. To achieve anoxic growth conditions, the medium was flushed with nitrogen gas (N_2_) for 5 min, where after the culture flask was immediately sealed airtight.

The bacterial growth of *P. aeruginosa* in the alginate beads was studied after 24 and 48 h in response to four permutations of the growth conditions: (i) Normoxic culture without NO_3_^−^, (ii) normoxic culture with NO_3_^−^, (iii) anoxic culture without NO_3_^−^, and (iv) anoxic culture with NO_3_^−^. Separate culture flasks were prepared for each time point, but all cultures originated from the same two batches of alginate beads (with and without NO_3_^−^) inoculated with the same PAO1 ON culture.

### Optical coherence tomography

We used a commercially available spectral-domain OCT system (Ganymede II, Thorlabs GmbH, Germany) equipped with an objective lens with an effective focal length of 36 mm, and a working distance of 25.1 mm (LSM03; Thorlabs GmbH, Germany). The operating principle and components of the OCT system are described in detail elsewhere^63,64^ and in Supplementary. In order to use the OCT signal to compare bacterial growth between experimental treatments, OCT measurements were performed under well-defined optical conditions in terms of OCT system settings. A single alginate bead was placed in a black screw cap filled with 800 µL of distilled water. Using *z*-axis OCT scans (so-called A-scans), the image was brought into focus via the manual focusing stage and by adjusting the reference light intensity as well as the position of the reference length.^64^ After optimisation of image acquisition settings, the configuration was not changed, and subsequent measurements on alginate beads were performed under identical conditions. Each alginate bead was first scanned at high resolution in three (technical) replicate cross-sectional scans (so-called B-scans) followed by one full 3D scan (so-called C-scan) and rendering of the entire bead. OCT imaging was done in (biological) triplicates for each treatment after 24 and 48 h of incubation, respectively.

Visualisation of OCT B-scan and C-scan was done with the manufacturers imaging software (ThorImage 4.2; Thorlabs GmbH, Germany) using the built-in brightness and contrast functions. The images were visualised assuming a constant refractive index of water (*n* = 1.33). The attenuation of the A-scan signal can be used to understand changes in the structure of the alginate bead. We extracted three vertical A-scans from the B-scans over the area surrounding the highest point of the alginate bead (Fig. S1). OCT images were extracted in B-scan mode with manually optimised brightness and contrast adjustment and assuming a refractive index of 1.33 (for water). These adjustments separated the background noise from the OCT signal generated from the alginate beads.

### Microscopy

A confocal laser scanning microscope (Zeiss Imager.Z2, LSM710 CLSM; Zeiss) operated with the manufacturers software (Zen2010, version 6.0; Zeiss, Germany) was used for imaging alginate-encapsulated, GFP-tagged PAO1. Samples were prepared by cutting the beads in half with a sterile scalpel. Cut beads were mounted in the dents of a flow-cell with the cut surface facing upwards. 10 µL milliQ water was applied to the cut bead surface, and a cover glass was fixed to the flow-cell with silicone sealant, making sure the cut surface was in close contact with the cover glass. The flow-cell was mounted on the microscope, and images were taken from the cut surface of the bead enabling visualisation of the distribution of bacterial aggregates from the periphery of the beads and towards the bead interior. Imaging of GFP-tagged *P. aeruginosa* was done with a 40×/NA1.3 oil objective, using laser excitation at 488 nm, and an emission filter range from 495 to 605 nm with a peak at 510 nm. The resulting images were processed with Imaris image processing software (v8.3.1; Bitplane, Switzerland).

### Microscopy combined with quantitative PNA-FISH

Alginate beads from the four permutations, sampled after 24 and 48 h, were stored for at least 24 h in 4% formaldehyde (Hounisen, Denmark) kept at 4 °C and supplemented with 0.25 CaCl_2_ for stabilisation. Subsequently, the beads were embedded in paraffin, cut in 4-µm thick sections with a standard microtome, fixed on cover slides and kept dark at 4 °C until further use; we note that this procedure did not work with the anoxic beads incubated without NO_3_^−^ due to bead disruption. Staining of bead sections was conducted with a Texas Red-conjugated PNA-FISH probe specific for *P. aeruginosa* 16S rRNA (AdvanDx, USA) by previously described methods.^14,21^ A microscope slide with the fixated and PNA-FISH stained 4-µm sections of alginate beads was mounted on the microscope. Imaging of the alginate bead sections for quantitative PNA-FISH was done with identical image acquisition settings for all pictures: Fluorescence images were recorded as 1-μm step size z-stacks at an image resolution of 4096 × 4096 pixels, with an averaging of two scans, and 16-bit colour depth using a 63×/1.4NA oil immersion objective, laser excitation at 594 nm, and emission range from 600 to 695 nm with a peak of 615 nm. Quantification of microcolony fluorescence (mean intensity) was performed using Imaris image processing software (v8.3.1; Bitplane, Switzerland). Image thresholding was applied, which discriminated background and foreground fluorescence with the use of the Measuring Pro expansion pack for the Imaris software (Bitplane, Switzerland). A minimum colony size was set to 10 µm^3^ to avoid inclusion of planktonic bacteria in the analysis of bacterial aggregates.^14^ The mean fluorescence intensity of each micro colony was measured on a 16-bit scale from 0–65535 fluorescence intensity units (FU). Kragh et al.^21^ previously described a linear correlation between growth rate and fluorescence intensity of PNA-FISH stained rRNA molecules in *P. aeruginosa*. Based on this relationship, the fluorescence intensity of PNA-FISH stained samples could be used as a proxy for apparent growth rate. Colony distance from the discernible periphery of alginate beads was measured manually for individual colonies using the measuring tool in the Imaris software (Bitplane, Switzerland) and was plotted against fluorescence intensity.

### Colony-forming units

For quantification of CFU, beads were dissolved using a solution of Na_2_CO_3_ (0.05 M) and citric acid (0.02 M).^65^ The dissolved bead slurry was serially diluted before plating on LB plates for enumeration of cells via colony formation. CFUs were determined in biological triplicates.

### Microsensor measurements

Beads were submerged in a Petri dish filled with pre-warmed R2A medium (with or without NO_3_^−^) that was kept at 37 °C under gentle ventilation by a fine air or nitrogen stream directed towards the surface via a Pasteur pipette connected to an air pump or N_2_ gas cylinder. Profiles of O_2_ concentration vs. depth in the alginate bead were measured with an amperometric O_2_ microsensor (25 µm tip diameter; OX25, Unisense A/S, Denmark) mounted on a motorised micromanipulator (MU1 Pyro-Science GmbH, Germany). Similarly, N_2_O concentration profiles were measured with an O_2_-insensitive amperometric N_2_O microsensor (25 µm tip diameter; N2O25, Unisense A/S, Denmark). All measurements were performed in 3−4 biological replicates. Both microsensors were connected to a pA metre (Unisense A/S, Denamark) that was interfaced to a PC via an A/D converter (Profix; Pyroscience GmbH, Germany). Linear calibrations of the microsensors were performed as specified by the manufacturer via measurements with the O_2_ microsensor in air saturated and O_2_-free water, and measurements with the N_2_O microsensor in N_2_O-free water, followed by measurement upon addition of defined aliquots of N_2_O saturated water.

Concentration profiles were recorded in beads incubated for 24 and 48 h after encapsulation of *P. aeruginosa* in alginate, respectively. The position, where the microsensor tip touched the bead surface (depth = 0), was determined visually with the aid of a USB microscope (model AM7515MZTL, dino-lite.eu) aiming after the centre of the uppermost bead surface. Profile measurements were conducted in steps of 50 or 200 µm through the bead. Microsensor positioning and data acquisition were done with dedicated profiling software (Profix; Pyro-Science GmbH, Germany).

To assess N_2_O concentration directly in the growth medium, the sensor tip was submerged in the growth medium and the resulting concentration was recorded as quickly as possible after removal of the cotton or rubber sealing.

### Calculations of O_2_ and N_2_O flux

Net production of N_2_O and net consumption of O_2_ in the alginate beads were estimated as gas fluxes from measured steady-state concentration profiles, by a modified version of Fick’s first law of diffusion,^66^ where the slope of the profile in the alginate bead was calculated from three consecutive measurements, from the upper quasi-linear parts of the profiles:^39^

1 $${{J}} = 0.5\left[ { - {{D}}\frac{{{{C}}_{\mathrm{a}} - {{C}}_{\mathrm{b}}}}{{{{X}}_{\mathrm{a}} - {{X}}_{\mathrm{b}}}}} \right] + 0.5\left[ { - {{D}}\frac{{{{C}}_{\mathrm{b}} - {{C}}_{\mathrm{c}}}}{{{{X}}_{\mathrm{b}} - {{X}}_{\mathrm{c}}}}} \right],$$

where *J* is the flux of O_2_ or N_2_O (nmol cm^−2^ min^−1^), *D* is the molecular diffusion coefficient of O_2_ (1.5 × 10^−5^ cm^2^ s^−1^)^55^ or N_2_O (2.76 × 10^−5^ cm^2 ^s^−1^)^67^ in water at 37 °C, and *C* is the concentration of O_2_ or N_2_O (µmol L^−1^) at depth *x*_*n*_ (µm), where *n* = a, b or c denotes three subsequent measurements at increasing depth (Eq. 1).

### NO_3_^−^ and NO_2_^−^ quantification

The NO_3_^−^ and NO_2_^−^ concentration in fresh and spent NO_3_^−^-supplemented R2A medium during the time course of the experiments was quantified by the Griess colorimetric reaction (no. 780001, Cayman Chemicals, USA), in technical duplicates, as previously described.^39^

### Imaging of O_2_

In order to image the O_2_ distribution within alginate beads harbouring *P. aeruginosa*, O_2_-sensitive sensor nanoparticles were incorporated into the beads. The sensor nanoparticles contained an O_2_-sensitive indicator (PtTFPP) and an insensitive reference dye (MY) and were prepared as described elsewhere.^33,68^ The sensor nanoparticles were added to the alginate-bacterial solution prior to bead preparation (sensor particle to alginate ratio: 1:50 vol/vol). After 24 h of growth, ratiometric O_2_ imaging of the bead was performed as described below.

For imaging, a bead was cut in half and placed on a cover glass with the cut surface facing the glass surface. The cut bead was covered with fresh medium and left to acclimate for a few minutes. A USB RGB microscope with built-in UV (405 nm) LED illumination (model AM4113-FVT, dino-lite.eu) was placed below the cover glass and was used to obtain images of the cut bead surface. After image acquisition, the obtained RGB images were split into the three colour channels (red, green, and blue) using the free image analysis software ImageJ (imagej.net). As the O_2_-sensitive indicator emits in the red channel and the reference indicator in the green channel, the ratio between these two channels could be used to obtain an O_2_ image. Ratios were calculated using the plugin Ratio Plus (https://imagej.nih.gov/ij/plugins/ratio-plus.html) and were linked to O_2_ concentration by a previously obtained calibration using the curve fitting function of ImageJ. For the calibration, alginate beads with sensor nanoparticles but without bacteria were submerged and imaged in water containing known O_2_ levels. The O_2_ levels were adjusted by flushing the water with an air/N_2_ mixture using a PC-controlled gas mixer (Sensorsense, The Netherlands). Detailed additional information on ratiometric O_2_ imaging using sensor nanoparticles can be found in recent publications.^32,33,68^

### Statistics

Data were analysed for statistical significance with SPSS version 24 (IBM, USA) and GraphPad Prism version 6 (GraphPad Software, USA). Data were illustrated with GraphPad Prism. Group comparisons for quantitative PNA-FISH data (Fig. 3) were made using either Mann–Whitney U test (for growth depth calculated as IQR) or independent samples *t*-test on log transformed values (apparent growth rate, expressed as mean fluorescence intensity). Log growth rate and depth were correlated using Pearson correlation. Flux rates (Fig. 5) were compared by Mann–Whitney U test. Changes in CFU (Fig. 4), concentrations of NO_3_^−^ and NO_2_^−^ (Fig. 6), as well as differences in OCT dB signal over time (Fig. 1) were analysed by two-way ANOVA. Based on our experience with the effect of treatment ​and the standard deviation of the methods rarely exceeding 40% of the effect we expect that three replicates will allow us to detect significant differences with *p* < 0.05 and a power of 0.80. A two-sided *α* of 0.05 was considered significant.

## Electronic supplementary material

Supplementary material
