# Supplementary material for: Tools for studying growth patterns and chemical dynamics of aggregated Pseudomonas aeruginosa exposed to different electron acceptors in an alginate bead model
Source: NPJ Biofilms Microbiomes. 2018 Feb 19;4:3. doi: 10.1038/s41522-018-0047-4 (PMC5818519; doi:10.1038/s41522-018-0047-4)
Supplement: Supplementary file 1 — Supplementary material [file 41522_2018_47_MOESM1_ESM.pdf]

Operating principles of OCT

The measuring principle of OCT is based on low coherence interferometry [1]. OCT measures directly backscattered photons from different reflective layers in a sample, e.g. at refractive index mismatches in a tissue. Signal contrast in OCT thus depends on refractive index fluctuations, where highly scattering materials create good signal contrast. Organic material scatter light due to spatial differences in e.g, protein and lipid density, which lead to microscopic fluctuations in the refractive index of cells and thus signal contrast in OCT. The high sensitivity of OCT to biological material has facilitated the non-invasive observation of human tissues, but has also found application in environmental microbiology, via the monitoring of e.g., biofilm formation [2].

While light scattering due to biological material creates signal contrast, it also reduces the vertical penetration depth of the OCT signal as light is lost due to scattering. Most OCT systems use light sources that employ near infrared radiation, which facilitates enhanced light penetration through biological tissues due to low light absorption. The vertical light attenuation of the OCT signal is thus primarily a function of 1) light scattering properties of the material, 2) the focus function of the objective and 3) the roll off response due to the spectral resolution of the spectrometer [3]. For our measurements, the latter two parameters were kept constant such that differences in light attenuation between the experimental treatments were only a function of the light scattering properties of the material.

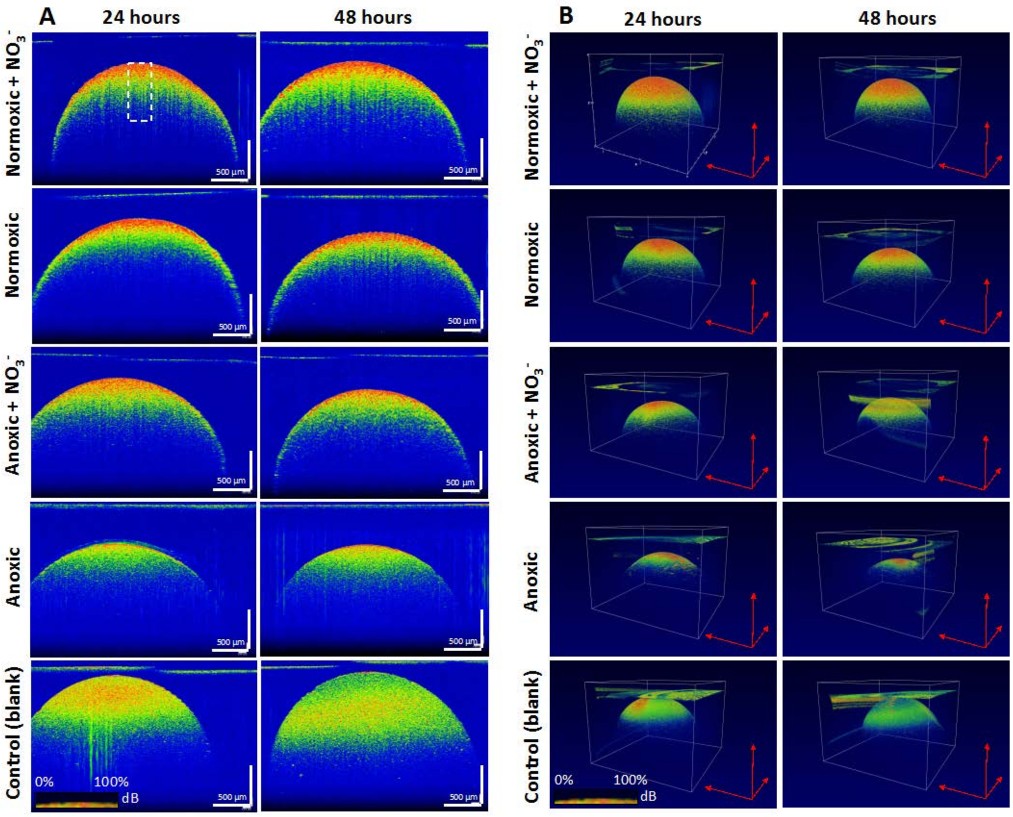

Figure S1. Optical coherence tomography A) Cross-sectional OCT B-scans visualising the growth dynamics of *P. aeruginosa* in alginate beads with different electron acceptors after 24 hours and 48 hours of incubation. The OCT B-scans were acquired in the centre of the alginate beads. The uncalibrated OCT dB signal is shown in false colour code and was normalised to the maximum OCT dB signal measured. The white rectangle shows an example of the image area that was used to calculate the average A-scan signal attenuation (Supplementary fig. S2) B) Three-dimensional rendering of the alginate beads shown in Panel A. OCT scans are shown in x,y,z dimensions. Red arrows are 1 mm in length in each dimension. Note that the planar surface above the alginate bead is an optical artefact related to the inference signal from the air-water interface.

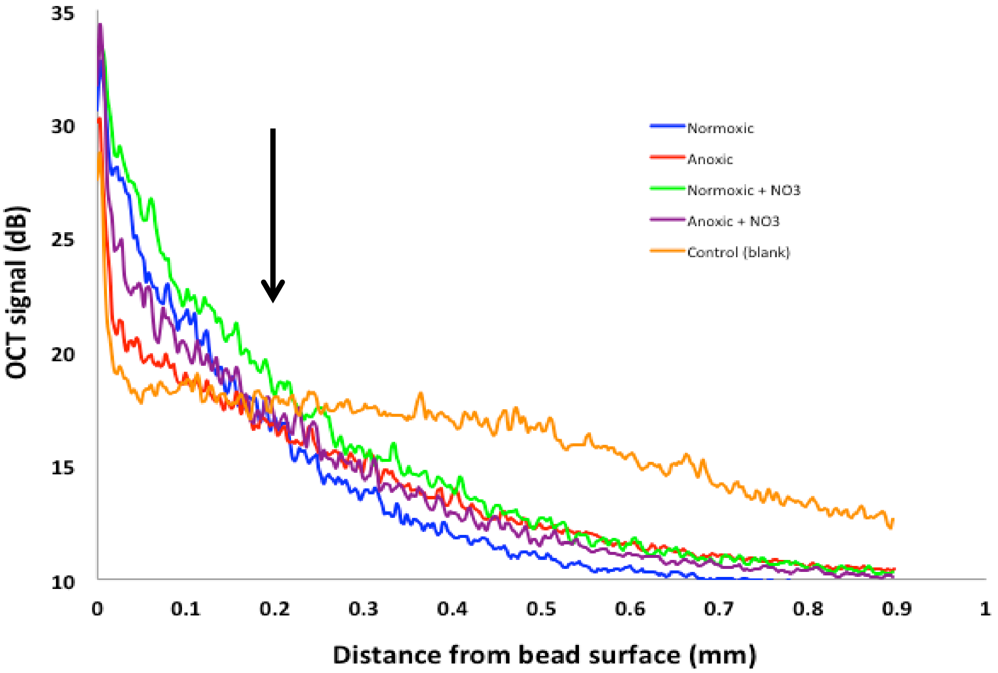

Figure S2. Vertical attenuation of the OCT signal for the different treatments. Data was averaged for a lateral width of 100 µm. The arrow indicates were the OCT signal approaches that of the OCT signal of pure alginate (control).

References

1 Fujimoto, J. G., Pitris, C., Boppart, S. A. & Brezinski, M. E. Optical coherence tomography: an emerging technology for biomedical imaging and optical biopsy. *Neoplasia* **2**, 9-25 (2000).

2 Xi, C. W., Marks, D., Schlachter, S., Luo, W. & Boppart, S. A. High-resolution three-dimensional imaging of biofilm development using optical coherence tomography. *J Biomed Opt* **11**, doi:10.1117/1.2209962 (2006).

3 Levitz, D. *et al.* Quantitative characterization of developing collagen gels using optical coherence tomography. *J Biomed Opt* **15**, 026019, doi:10.1117/1.3377961 (2010).
